# Chromosomal abnormalities in spontaneous abortion after assisted reproductive treatment

**DOI:** 10.1186/1471-2350-11-153

**Published:** 2010-11-03

**Authors:** Ji Won Kim, Woo Sik Lee, Tae Ki Yoon, Hyun Ha Seok, Jung Hyun Cho, You Shin Kim, Sang Woo Lyu, Sung Han Shim

**Affiliations:** 1 Department of Obstetrics and Gynecology, Fertility Center of CHA Gangnam Medical Center, CHA University, Seoul, Korea; 2 Genetic Laboratory, Fertility Center of CHA Gangnam Medical Center, CHA University, Seoul, Korea

## Abstract

**Background:**

We evaluated cytogenetic results occurring with first trimester pregnancy loss, and assessed the type and frequency of chromosomal abnormalities after assisted reproductive treatment (ART) and compared them with a control group. We also compared the rate of chromosomal abnormalities according to infertility causes in ICSI group.

**Methods:**

A retrospective cohort analysis was made of all patients who were referred to the Genetics Laboratory of Fertility Center of CHA Gangnam Medical Center from 2005 to 2009 because of clinical abortion with a subsequent dilation and evacuation (D&E) performed, and patients were grouped by type of conception as follows: conventional IVF (*in vitro *fertilization) (n = 114), ICSI (intracytoplasmic sperm injection) (n = 140), and control (natural conception or intrauterine insemination [IUI]) (n = 128). Statistical analysis was performed using SPSS software.

**Results:**

A total 406 specimens were referred to laboratory, ten abortuses were excluded, and in 14 cases, we did not get any spontaneous metaphase, chromosomal constitutions of 382 specimens were successfully obtained with conventional cytogenetic methods. Overall, 52.62% of the miscarriages were found to be cytogenetically abnormal among all patients, the frequency was 48.4% in the control group, 54.3% of miscarriages after ICSI and 55.3% after conventional IVF (p = 0.503). The most prevalent abnormalities were autosomal trisomy, however, nine (11.69%) sex chromosome aneuploidy were noted in the ICSI group vs. four (6.45%) and two (3.23%) cases in the conventional IVF group and control group. We compared chromosomal abnormalities of miscarriages after ICSI according to infertility factor. 55.71% underwent ICSI due to male factors, 44.29% due to non-male factors. ICSI group having male factors showed significantly higher risk of chromosomal abnormalities than ICSI group having non-male factors (65.8% vs. 34.2%, *p *= 0.009, odds ratio = 1.529, 95% CI = 1.092-2.141).

**Conclusions:**

There is no increased risk of chromosomal abnormalities due to ART was found with the exception of a greater number of sex chromosomal abnormalities in the ICSI group with male factor infertility. Therefore, these alterations could be correlated with the underlying parental risk of abnormalities and not with the ICSI procedure itself.

## Background

Spontaneous abortion occurs in 15-20% of clinically recognized gestations [1]. Genetic defects, especially chromosomal abnormalities, are the most common cause of spontaneous miscarriage during the first trimester; indeed, chromosomal abnormalities occur in approximately 60% of such cases [2]. It is unclear whether pregnancies conceived through assisted reproductive treatment (ART) are at an increased risk of pregnancy loss compared with naturally conceived pregnancies, when accounting for maternal age and ART procedures [3-5].

Few reports have examined pregnancy outcomes after ART, and their results are inconclusive with regards to the risk of miscarriage and aneuploidy. Intracytoplasmic sperm injection (ICSI) is associated with an increased incidence of aneuploidy and *de novo *sex chromosome aberrations [6-8]. The additional risk of chromosomal abnormalities for children conceived through ICSI is approximately 1% above baseline, which is small but not negligible, and a prenatal diagnosis may be offered [9]. Additionally, some studies found that major congenital malformations were more frequent among ICSI offspring [10,11]. However, several studies report conflicting results. Specifically, one study reported no increased risk of congenital malformation or major developmental delays associated with ICSI [12], and another showed that couples that underwent ART did not exhibit increased cytogenetic risks compared to a natural conception group, and no significant difference was seen in the incidence of chromosomal anomalies between ICSI and *in vitro *fertilization (IVF) [13].

Few data from large sample sizes have been reported on chromosomal abnormalities in miscarriages after ART [13-15]. Thus, it remains controversial whether ART results in an increased risk of chromosomal abnormalities resulting in a first trimester miscarriage. The aim of this study was to evaluate the types and frequencies of chromosomal abnormalities occurring after different types of ART and to compare the outcomes for each ART group to those for a control group. Moreover, we compared the chromosomal abnormalities in miscarriages after ICSI according to infertility factors.

## Methods

A retrospective cohort analysis was conducted using patients who were referred to the Genetics Laboratory of the Fertility Center of CHA Gangnam Medical Center from 2005 to 2009 following clinical abortion with subsequent dilatation and evacuation (D&E). Patients were grouped by the type of conception: conventional IVF, ICSI, and controls, which included nonviable spontaneous pregnancies from infertile couples who had not undergone ART and resulting from intrauterine insemination (IUI).

Except natural cycle protocol such as *in vitro *maturation (IVM), most patients used either the GnRH agonist (Lucrine^®^; Abbott, Cedex, France) long or GnRH antagonist (Cetrotide^®^; Serono, Geneva, Switzerland) protocol for controlled ovarian stimulation (COS) along with daily injections of rFSH (Gonal-F^®^; Serono or Puregon^®^; Organon, Oss, The Netherlands). And fertilization underwent, either by conventional insemination or ICSI in IVF medium (Quinn's Advantage Medium^®^; SAGE BioPharma, Bedminster, NJ). Fresh ejaculated sperm were evaluated by standard andrological screening and World Health Organization (WHO) values (volume, count, and motility); Kruger morphologies were utilized to interpret semen quality [16]. In addition, testicular sperm extraction (TESE) was performed for patients with azoospermia or severe oligoathenozoospermia (OAT).

Samples were obtained by D&E after spontaneous abortion and collected in sterile containers containing 10 ml of RPMI-1640 with L-glutamine (Invitrogen, CA, USA). In the Genetics Laboratory, trained technologists dissected and selected the placental chorionic villi and cultured them in BIO-AMP-2 complete medium (Biological Industries Ltd., Haemek, Israel) at 37°C in a 5% CO_2 _incubator. All procedures, including cell harvesting, slide preparation, and staining, were conducted following standard protocols [17]. At least 20 GTG-banded metaphases were analyzed in each case.

Statistical analysis was performed using Statistical Program for Social Science (SPSS 13.0, Chicago, IL) software. Continuous variables were reported as the mean ± standard deviation or as the median and range, depending on their distribution, with a normal distribution defined using the one-way ANOVA test. Between-group comparisons of normally distributed variables were assessed using Student's *t*-test. Categorical variables were compared using the chi-square test. The significance level for all analyses was set at *p *< 0.05. All data are reported as means with their associated standard deviations. Odds ratios and 95% confidence intervals (CIs) are shown where appropriate.

The study protocol was approved by the Institutional Review Board of CHA Gangnam Medical Center.

## Results

A total of 406 specimens were assessed. Ten abortuses were excluded for the following reasons: one was terminated due to fetal anomalies during the second trimester, two were diagnosed as hydatidiform moles, and seven cases involved a parental chromosomal abnormality. Four cases were conceived by preimplantation genetic diagnosis (PGD), while in fourteen out of 406 cases, cell culture failed because of absent or insufficient villi, no growth, or bacterial contamination after incubation. The chromosomal constitutions of 382 specimens were successfully obtained using conventional cytogenetic methods. Of these abortuses, 114 were derived from conventional IVF, 140 were derived from ICSI, and 128 were derived from a control group.

The mean age of the female spouse was 35.23 (range 34.81-35.65 years). The mean age of the male spouse was 37.2 (range 36.72-37.69 years). The patients' demographics, semen parameter results, basal hormonal profile, and fetal gestational age at D&E are shown in Table 1. The study population's demographics were similar for each group evaluated. However, as expected, paternal age, previous pregnancy status, and semen parameters varied significantly. Seminal volume did not differ in each group. However, sperm count, morphology, and motility were significantly decreased in the ICSI group, whereas no differences were seen between the control and conventional IVF groups. The duration of infertility was 5.28 ± 5.96 years in the conventional IVF group and 4.89 ± 3.28 years in the ICSI group (*p *= 0.521). In the conventional IVF group, 56.1% (46/82) underwent the GnRH agonist long protocol while 43.9% (36/82) underwent the GnRH antagonist protocol for COS, whereas in the ICSI group, the numbers were 46.94% (46/98) and 53.06% (52/98), respectively. The mean number of retrieved oocytes, mature oocytes, fertilized oocytes, embryos cultured on day 3, and transferred embryos were not significantly different between the two groups. The mean number of good-quality embryos or embryos transferred (ET), however, was 2.28 ± 1.01 in the conventional IVF group and 1.66 ± 1.12 in the ICSI group (*p *< 0.001).

**Table 1:** Patient demographics and laboratory findings

	Control group (n = 128)	Conventional IVF group (n = 114)	ICSI group (n = 140)	p-value
Maternal age (yr)	34.52 ± 4.13	35.70 ± 3.97	35.49 ± 4.23	0.055
Paternal age (yr)	36.05 ± 4.34^a^	37.43 ± 4.39^a,b^	38.07 ± 5.40^b^	0.002
Maternal BMI	21.13 ± 2.49	21.16 ± 3.04	21.73 ± 2.93	0.162
Semen analysis				
Volume (ml)	2.91 ± 1.59	2.83 ± 1.18	2.57 ± 1.42	0.167
Count (x10^6^/ml)	91.85 ± 43.72 ^a^	93.62 ± 58.41 ^a^	66.48 ± 66.50 ^b^	< 0.001
Morphology, strict	6.14 ± 2.36 ^a^	6.68 ± 2.86 ^a^	4.25 ± 3.57 ^b^	< 0.001
Motility	47.90 ± 15.46 ^a^	48.56 ± 16.75 ^a^	37.99 ± 21.11 ^b^	< 0.001
Hormonal profile				
Basal FSH (u/ml)	7.17 ± 2.76	7.49 ± 5.08	7.28 ± 3.58	0.886
Basal LH (u/ml)	4.18 ± 4.01	3.06 ± 1.71	3.83 ± 3.08	0.127
Basal E2 (pg/ml)	28.25 ± 14.29	28.41 ± 21.74	30.44 ± 24.19	0.729
Fetal gestational age at D&E (days)	59.66 ± 8.23	61.00 ± 7.51	60.98 ± 7.77	0.300
Previous pregnancy status (n)				
Parity	1.63 ± 1.47^a^	1.32 ± 1.51^a,b^	0.99 ± 1.18^b^	0.001
Abortion	1.11 ± 1.21^a^	0.61 ± 0.98^b^	0.49 ± 0.66^b^	< 0.001

Yr: years, BMI: body mass index, D&E: dilatation and evacuation, FSH: follicular stimulating hormone, LH: luteinizing hormone, E2: estradiol, IVF: in vitro fertilization, ICSI: intracytoplasmic insemination

Values are mean ± SD

Values with different superscripts are significantly different between ^a ^and ^b ^(p < 0.05).

Values with same superscripts are no significantly different between ^a ^and ^a ^or ^b ^and ^b^.

Overall, 52.62% (201/382) of the miscarriages were found to be cytogenetically abnormal among the patients, with 48.44% (62/128) in the control group, 54.29% (76/140) after ICSI, and 55.26% (63/114) after conventional IVF; there were no statistically significant differences (*p *= 0.503). Specifically, 62.5% (55/88) of miscarriages occurred after IVF-fresh cycle, 30.77% (8/26) following IVF-thawing embryo transfer (TET), 56.44% (57/101) from ICSI-ejaculated sperm, 66.67% (6/9) after ICSI-TESE, 60% (9/15) following ICSI-TET, and 26.67% (4/15) after IVM-ICSI (Table 2).

**Table 2:** Normal vs. abnormal karyotypes of miscarriages according to conception methods

	Normal			Abnormal			Total
		
	Male factor	Non-male factor	Total	Male factor	Non-male factor	Total	
Total ICSI	28 (43.75%)	36 (56.25%)	64 (45.71%)	50 (65.79%)^b^	26 (34.21%)^b^	76 (54.29%)^a^	140
ICSI, ejaculated	19	25	44 (43.56%)	37	20	57 (56.44%)	101
ICSI, TESE	3	-	3 (33.33%)	6	-	6 (66.67%)	9
ICSI-TET	5	1	6 (40%)	5	4	9 (60%)	15
IVM	1	10	11 (73.33%)	2	2	4 (26.67%)	15
Total conventional IVF	51 (44.7%)			63 (55.26%)^a^			114
IVF-fresh	33			55			88
IVF-TET	18			8			26
Control	66 (51.56%)			62 (48.44%)^a^			128
Total	181 (47.38%)			201 (52.62%)			382

TESE: testicular sperm extraction, TET: thawing embryo transfer, IVM: in vitro maturation

^a^p = 0.503

^b^p = 0.009, OR = 1.529 (95% CI = 1.092 - 2.141)

We compared the chromosomal abnormalities in miscarriages after ICSI according to infertility factors. A total of 55.71% (78/140) underwent ICSI due to male factors and 44.29% (62/140) due to non-male factors. The ICSI group with male factors showed a significantly increased risk of chromosomal abnormalities compared to the ICSI group with non-male factors (65.79 vs. 34.21%, *p *= 0.009, odds ratio = 1.529, 95% CI = 1.092-2.141) (Table 2).

We summarizes the distribution of cytogenetic results for all of the pregnancies examined in the study. The distribution of cytogenetic results was similar; however, the incidence of sex chromosome aneuploidy was 11.69% (9/77) in the ICSI group vs. 6.45 (4/62) and 3.23% (2/62) in the conventional IVF and control groups, respectively. If we add the one observed instance of mosaic of sex chromosomes and two combined sex and autosomal chromosome aneuploidies, the frequency is increased by 15.58% (12/76) compared with non-ICSI pregnancies, including conventional IVF and the control group (7.2%, 9/125) (*p *= 0.054). This, however, was not statistically significant, although it might be due to the small size. In the ICSI-TESE group, only autosomal trisomies were found. The most prevalent abnormalities observed in first trimester pregnancy loss were autosomal aneuploidy (79.6%; 160/201), similar to the ICSI and conventional IVF and control groups (77.6, 77.8, and 83.9%, respectively) (*p *= 0.604). Autosomal trisomy was the most common aneuploidy. In the ART group (including conventional IVF and ICSI), trisomy 22 (24.24%, 24/99) was the most frequent anomaly, followed by trisomy 16 (18.18%, 18/99); 15 (10.1%, 10/99); 4, 7, 8, and 21 (5.05%, 5/99 each); 20 (4.04%, 4/99); 3, 5, 12, 13, and 14 (3.03%, 3/99 each); 9 and 18 (2.02%, 2/99 each); and 2, 6, 10, and 11 (each was found in only one case, 1.01%). In the control group, trisomy 16 and 22 (20%, 10/50 each) were the most common, followed by trisomy 15 (12%, 6/50); 7 and 13 (8%, 4/50 each); and 2, 8, 10, 12, and 20 (4%, 2/50 each). Double aneuploidy (including double autosomal aneuploidy and combined autosomal and sex chromosome aneuploidy) occurred in 7.91% (11/139) of cases in the ART group and 4.84% (3/62) of cases in the control group. Triple aneuploidy occurred in one only of the ART cases. Polyploidy was observed in 6.47% (9/139) of the ART cases and 9.68% (6/62) of the control group cases.

Among the miscarriages with normal cytogenetic results, 37.57% (68/181) were found to be normal male and 62.43% (113/181) were found to be normal female karyotypes. Among the miscarriages with abnormal cytogenetic abnormalities, 55.22% (111/201) were found to be abnormal female (without Y chromosome) while 44.78% (90/201) were found to be abnormal male karyotypes (with Y chromosome).

## Discussion

We found no significant increase in the occurrence of chromosomal abnormalities in early pregnancy loss when conception occurred through ART when compared with the control group. However, sex chromosomal abnormalities were increased among pregnancies resulting from ICSI. Moreover, the ICSI group with male factors showed a significantly increased risk of chromosomal abnormalities compared to the ICSI group with non-male factors.

Concerns about ICSI-related chromosomal aberrations were first raised in 1995 [18,19]. In't Veld *et al*. [19] first reported that 33% of ICSI pregnancies, in which all five chromosomal abnormalities were sex chromosomal nature, identified by prenatal diagnosis. However, their study included a selection bias, as it was based on a referral for advanced maternal age for prenatal diagnosis. Additionally, the sample size was small and there was no description of the parents' genetic status. Nevertheless, the relatively high risk of aneuploidy led to an expanded study [18], which identified chromosomal anomalies in 1% of cases, and five of the six abnormalities reported involved sex chromosomes. Several other investigators followed up on these initial reports, with some subsequent reports suggesting an increased rate of chromosomal aberrations in ICSI conception [6,8,9,20]. ICSI bypasses natural selection mechanisms and may increase first trimester aneuploidy rates [6]. Explanations for this include (I) physical or biochemical disturbances of the ooplasma or meiotic spindle, (II) injection of biochemical contaminants, (III) injection of sperm-associated exogenous DNA, (IV) injection of sperm carrying a chromosomal anomaly, (V) transmission of genetic defects that may be related to the underlying male-factor infertility, (VI) male gametes with structural defects, (VII) anomalies of sperm activating factors, (VIII) potential for incorporating sperm mitochondrial DNA, and (IX) female gamete anomalies [7]. However, other studies of the incidence of aneuploidy in normal fetuses and abortuses after IVF or ICSI produced conflicting results [12,13]. Our study, which included a large sample size, confirmed no significant difference for the total abnormal karyotype rate in the abortuses between the two groups (54.3% for ICSI and 55.3% for conventional IVF), and there were no significant differences compared with the control group (48.4%) (*p *= 0.503).

We assessed the type and frequency of chromosomal abnormalities following different ART treatments and compared them with those following natural conception. Among the normal cytogenetic results, a normal female karyotype was found 1.7 times more frequently than a normal male karyotype. However, the sex ratio among abortuses with chromosomal anomalies did not differ, similar to previous studies [7]. In particular, sex ratios among the abnormal cytogenetic results were 0.8 in the conventional IVF group, 0.85 in the ICSI group, and 0.77 in the control group. The sex ratio discrepancy among the normal cytogenetic results may be due to maternal cell contamination.

The most common abnormal karyotype encountered was autosomal trisomy in all groups, and the frequency was similar in each group. However, sex chromosome aneuploidy was observed more frequently among pregnancies resulting from ICSI compared to conventional IVF, in accordance with recent studies [7,15]. Several studies have documented a significant rise in the frequency of sex chromosomal abnormalities in sperm from males with abnormal spermatogenesis. Infertile men with a normal karyotype and abnormal spermatogenesis have been shown to carry a significantly increased risk of producing aneuploid spermatozoa, particularly for the sex chromosomes [12,21-23]. These studies are in accordance with the increased incidence of sex chromosomal abnormalities in the ICSI group. However, the causes remain independent of the ICSI process itself.

In previous reports, some authors noted that chromosomal anomalies occurred more often in those with severe indices, implicating the parental factor rather than the ICSI procedure [8,24]. Bunduelle et al. [8] suggested that the increased rate of aneuploidy observed was most likely related to a higher rate of aneuploidy in the sperm of the fathers, while Calogero et al. [25] demonstrated that patients with abnormal sperm parameters had an increased aneuploidy rate that was negatively correlated with sperm concentration and the percentage of normal sperm. A normal paternal karyotype does not exclude the possibility of germ cell aneuploidy because an altered intratesticular environment not only damages spermatogenesis but also disrupts the mechanisms controlling chromosomal segregation during meiosis [25]. In support of the suggestion of male-bearing chromosomal anomalies, we analyzed the ICSI group according to infertility causes (i.e., male and non-male factors). Male factors included azoospermia and OAT, while all others were defined as non-male factors. The ICSI group with male factors showed an approximately 1.5 times greater risk of chromosomal abnormalities compared to the ICSI group with non-male factors, indicating paternal origin of the abortuses with detected chromosomal aberrations after ICSI. In particular, abortuses with sex chromosomal abnormalities including numerical and structural anomalies derived from male factors were much more frequent than those with non-male factors (18 vs. 11.5%).

## Conclusions

We conducted a relatively large-scale study with separate subgroups analyzed according to different etiologies of infertility. Our results indicate that there is no risk increased chromosomal abnormalities due to ART, with the exception a greater number of chromosomal abnormalities in the ICSI group with male factor infertility. Therefore, these alterations could be correlated with the underlying parental risk of abnormalities and not with the ICSI procedure itself.

## Competing interests

The authors declare that they have no competing interests.

## Authors' contributions

JWK has made contributions to conception and design or acquisition of data and wrote the manuscript. HHS, YSK and JHC have made contributions to acquisition of data. WSL and TKY revised the manuscript critically for important intellectual content. SWL has made contributions to acquisition of date and performed the statistical analyses and interpretation of data. SHS has made contributions to conception, design, and revised it critically for important intellectual content. All authors read and approved final manuscript.

## Pre-publication history

The pre-publication history for this paper can be accessed here:

http://www.biomedcentral.com/1471-2350/11/153/prepub
